# The Melting Point of Phosphorus

**Published:** 1846-11

**Authors:** 


*The melting point of Phosphorus.*—M. Desains has lately made some experiments on this subject. According to him, most chemical writers state that phosphorus melts at 109° (104°?). In order to determine the temperature of fusion, the phosphorus must be melted under a thin stratum of water; a thermometer should then be plunged into the midst of the melted substance, and when the instrument stands at about 109°, it should be well agitated. The phosphorus then solidifies, and the temperature always rises to and remains at one uniform degree, which is the period of fusion and solidification. If the melted phosphorus were not stirred about, its temperature might fall twenty or thirty degrees lower; and then the latent heat evolved during solidification would not be sufficient to make it regain the true melting point. The degree at which the thermometer would remain stationary would depend on that to which the phosphorus had fallen before solidifying, and on the radiation of heat to the surrounding medium. The greater the quantity of latent heat taken by this medium, the less will remain to raise the temperature of the solid. M. Desains has found as the result of his experiments, that the temperature at which distilled phosphorus melts and becomes solid is 42°.2 centigrade. This is already equal to 112° F., a temperature much higher than that which has been hitherto assigned by chemists.—*Ib., from Comptes Rendus.*

